# (*E*)-2-Bromo-1-[2-(2-nitro­styr­yl)-1-phenyl­sulfonyl-1*H*-indol-3-yl]ethanone

**DOI:** 10.1107/S1600536814000488

**Published:** 2014-01-15

**Authors:** J. Kanchanadevi, G. Anbalagan, V. Saravanan, A. K. Mohanakrishnan, B. Gunasekaran, V. Manivannan

**Affiliations:** a Department of Physics, Velammal Institute of Technology, Panchetty, Chennai 601 204, India; b Department of Physics, Presidency College (Autonomous), Chennai 600 005, India; c Department of Organic Chemistry, University of Madras, Guindy Campus, Chennai 600 025, India; d Department of Physics & Nano Technology, SRM University, SRM Nagar, Kattankulathur, Kancheepuram Dist, Chennai 603 203 Tamil Nadu, India; e Department of Research and Development, PRIST University, Vallam, Thanjavur 613 403, Tamil Nadu, India

## Abstract

In the title compound C_24_H_17_BrN_2_O_5_S, the phenyl ring makes dihedral angles of 85.4 (2) and 8.8 (2)° with the indole ring system and the nitro­benzene ring, respectively, while the indole ring system and nitrobenzene ring make a dihedral angle of 80.1 (2)°. In the crystal, weak C—H⋯O inter­actions link the mol­ecules, forming a two-dimensional network parallel to the *bc* plane.

## Related literature   

For the biological activity of indole derivatives, see: Andreani *et al.* (2001 ▶); Singh *et al.* (2000 ▶); Pomarnacka & Kozlarska-Kedra (2003 ▶); Srivastava & Pandeya (2011 ▶). For a related structure, see: Umadevi *et al.* (2013 ▶); Kanchanadevi *et al.* (2013 ▶).
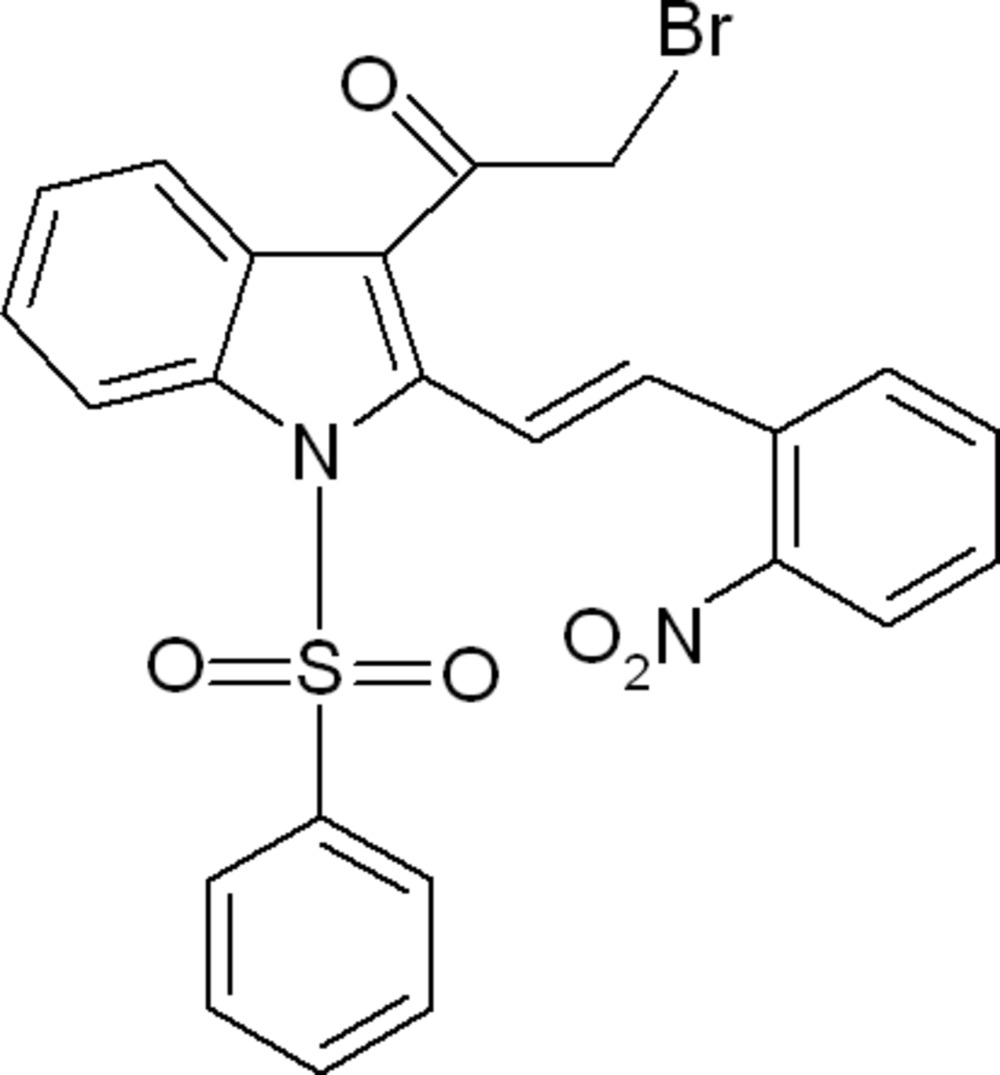


## Experimental   

### 

#### Crystal data  

C_24_H_17_BrN_2_O_5_S
*M* *_r_* = 525.37
Monoclinic, 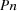

*a* = 10.1823 (7) Å
*b* = 8.0932 (6) Å
*c* = 13.8111 (12) Å
β = 102.749 (2)°
*V* = 1110.08 (15) Å^3^
*Z* = 2
Mo *K*α radiation
μ = 1.99 mm^−1^
*T* = 295 K
0.35 × 0.25 × 0.25 mm

#### Data collection  

Bruker APEXII CCD diffractometer
Absorption correction: multi-scan (*SADABS*; Sheldrick, 1996 ▶) *T*_min_ = 0.543, *T*_max_ = 0.609
17011 measured reflections
4323 independent reflections
3368 reflections with *I* > 2σ(*I*)
*R*_int_ = 0.027

#### Refinement  

*R*[*F* ^2^ > 2σ(*F* ^2^)] = 0.044
*wR*(*F* ^2^) = 0.116
*S* = 1.01
4323 reflections
298 parameters
2 restraints
H-atom parameters constrained
Δρ_max_ = 0.85 e Å^−3^
Δρ_min_ = −0.43 e Å^−3^
Absolute structure: Flack (1983 ▶), 2093 Friedel pairs
Absolute structure parameter: 0.022 (9)

### 

Data collection: *APEX2* (Bruker, 2008 ▶); cell refinement: *SAINT* (Bruker, 2008 ▶); data reduction: *SAINT*; program(s) used to solve structure: *SHELXS97* (Sheldrick, 2008 ▶); program(s) used to refine structure: *SHELXL97* (Sheldrick, 2008 ▶); molecular graphics: *PLATON* (Spek, 2009 ▶); software used to prepare material for publication: *SHELXL97*.

## Supplementary Material

Crystal structure: contains datablock(s) I. DOI: 10.1107/S1600536814000488/hg5374sup1.cif

Structure factors: contains datablock(s) I. DOI: 10.1107/S1600536814000488/hg5374Isup2.hkl

Supporting information file. DOI: 10.1107/S1600536814000488/hg5374Isup3.cml

CCDC reference:

Additional supporting information:  crystallographic information; 3D view; checkCIF report

## Figures and Tables

**Table 1:** Hydrogen-bond geometry (Å, °)

*D*—H⋯*A*	*D*—H	H⋯*A*	*D*⋯*A*	*D*—H⋯*A*
C2—H2⋯O5^i^	0.93	2.38	3.147 (5)	139

Symmetry code: (i) 
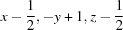
.

**Fig. 1.**
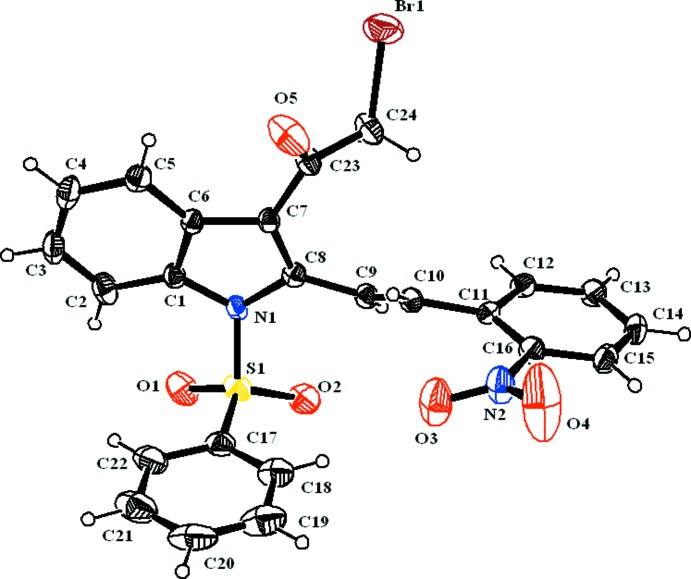
The molecular structure of (I), with atom labels and 30% probability displacement ellipsoids for non-H atoms.

**Fig. 2.**
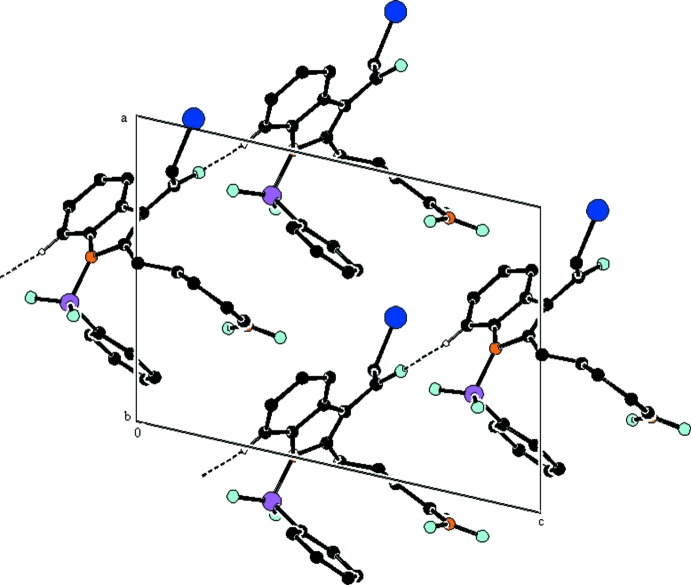
The packing of (I), viewed down the b axis. Hydrogen bonds are shown as dashed lines.

## supplementary crystallographic information

### 1. Comment

Indole derivatives exhibits antibacterial, antifungal (Singh *et al.*, 2000) and antitumour activities (Andreani *et al.*, 2001). These derivatives also exhibits antimicrobial, antibiotic, analgesic, anticancer and anti-HIV (Pomarnacka & Kozlarska-Kedra, 2003; Srivastava & Pandeya, 2011) activities.

The geometric parameters of the title molecule (Fig. 1) agree well with reported similar structure (Umadevi *et al.*, 2013). The phenyl ring makes a dihedral angles of 85.4 (2)° and 8.8 (2)° with the indole ring system and the nitro benzene ring, respectively. The sum of bond angles around the atom N1 [355.7°] indicates *sp^2^* hybridization. The molecular structure is stabilized by weak intramolecular C—H···O interactions and the crystal packing is controlled by weak intermolecular C—H···O interaction.

### 2. Experimental

A solution of 2-nitrophenylvinyl-3-acetylindole (1.0 g, 2.29 mmol) and PTT (Phenyl trimethyl ammonium tribromide) (0.92 g, 2.46 mmol) in dry THF (10 ml) was stirred at 10 ° C for 1 h. After completion of the reaction (monitored by TLC), it was poured into crushed ice (100 g). The solid obtained was filtered and washed with MeOH (5 ml) to afford (*E*)-2-bromo-1-(2-(2-nitrostyryl)-1-(phenylsulfonyl)-1*H*- indol-3-yl)ethanone as a yellow solid (0.92 g, 78%) with melting point 184–186 ° C.

### 3. Refinement

H atoms were positioned geometrically and refined using riding model with C—H = 0.93Å and *U*_iso_(H) = 1.2Ueq(C) for aromatic C—H, C—H = 0.97 Å and *U*_iso_(H) = 1.2Ueq(C) for CH_2_.

### Crystal data

**Table:**

C_24_H_17_BrN_2_O_5_S	*F*(000) = 532
*M**_r_* = 525.37	*D*_x_ = 1.572 Mg m^−^^3^
Monoclinic, *P**n*	Mo *K*α radiation, λ = 0.71073 Å
Hall symbol: P -2yac	Cell parameters from 4350 reflections
*a* = 10.1823 (7) Å	θ = 2.3–26.3°
*b* = 8.0932 (6) Å	µ = 1.99 mm^−^^1^
*c* = 13.8111 (12) Å	*T* = 295 K
β = 102.749 (2)°	Block, yellow
*V* = 1110.08 (15) Å^3^	0.35 × 0.25 × 0.25 mm
*Z* = 2	

### Data collection

**Table:**

Bruker APEXII CCD diffractometer	4323 independent reflections
Radiation source: fine-focus sealed tube	3368 reflections with *I* > 2σ(*I*)
Graphite monochromator	*R*_int_ = 0.027
Detector resolution: 0 pixels mm^-1^	θ_max_ = 26.3°, θ_min_ = 2.3°
ω and φ scans	*h* = −12→12
Absorption correction: multi-scan (*SADABS*; Sheldrick, 1996)	*k* = −9→9
*T*_min_ = 0.543, *T*_max_ = 0.609	*l* = −17→17
17011 measured reflections	

### Refinement

**Table:**

Refinement on *F*^2^	Secondary atom site location: difference Fourier map
Least-squares matrix: full	Hydrogen site location: inferred from neighbouring sites
*R*[*F*^2^ > 2σ(*F*^2^)] = 0.044	H-atom parameters constrained
*wR*(*F*^2^) = 0.116	*w* = 1/[σ^2^(*F*_o_^2^) + (0.0621*P*)^2^ + 0.3412*P*] where *P* = (*F*_o_^2^ + 2*F*_c_^2^)/3
*S* = 1.01	(Δ/σ)_max_ < 0.001
4323 reflections	Δρ_max_ = 0.85 e Å^−^^3^
298 parameters	Δρ_min_ = −0.43 e Å^−^^3^
2 restraints	Absolute structure: Flack (1983), 2093 Friedel pairs
Primary atom site location: structure-invariant direct methods	Absolute structure parameter: 0.022 (9)

### Fractional atomic coordinates and isotropic or equivalent isotropic displacement parameters (Å^2^)

**Table:**

	*x*	*y*	*z*	*U*_iso_*/*U*_eq_	
C1	0.0805 (4)	0.3950 (5)	0.3857 (3)	0.0358 (8)	
C2	0.0594 (5)	0.5284 (5)	0.3192 (3)	0.0516 (11)	
H2	−0.0157	0.5329	0.2669	0.062*	
C3	0.1535 (5)	0.6512 (5)	0.3345 (4)	0.0556 (11)	
H3	0.1419	0.7411	0.2915	0.067*	
C4	0.2643 (5)	0.6461 (5)	0.4112 (3)	0.0516 (11)	
H4	0.3264	0.7319	0.4184	0.062*	
C5	0.2867 (5)	0.5171 (5)	0.4783 (3)	0.0437 (10)	
H5	0.3617	0.5156	0.5309	0.052*	
C6	0.1926 (4)	0.3890 (4)	0.4643 (3)	0.0327 (8)	
C7	0.1862 (4)	0.2356 (4)	0.5157 (3)	0.0307 (7)	
C8	0.0699 (4)	0.1549 (4)	0.4711 (3)	0.0332 (8)	
C9	0.0184 (4)	0.0034 (4)	0.5039 (3)	0.0353 (8)	
H9	−0.0185	−0.0765	0.4574	0.042*	
C10	0.0230 (4)	−0.0236 (4)	0.5997 (3)	0.0340 (8)	
H10	0.0499	0.0638	0.6432	0.041*	
C11	−0.0110 (3)	−0.1803 (5)	0.6424 (3)	0.0330 (8)	
C12	0.0184 (4)	−0.3288 (4)	0.6024 (3)	0.0366 (9)	
H12	0.0557	−0.3276	0.5467	0.044*	
C13	−0.0058 (4)	−0.4776 (5)	0.6424 (3)	0.0445 (10)	
H13	0.0152	−0.5753	0.6138	0.053*	
C14	−0.0612 (5)	−0.4832 (5)	0.7250 (4)	0.0483 (11)	
H14	−0.0772	−0.5844	0.7522	0.058*	
C15	−0.0924 (4)	−0.3399 (5)	0.7662 (3)	0.0462 (10)	
H15	−0.1307	−0.3427	0.8214	0.055*	
C16	−0.0671 (4)	−0.1910 (5)	0.7261 (3)	0.0391 (9)	
C17	−0.2379 (4)	0.3680 (6)	0.4081 (3)	0.0478 (11)	
C18	−0.2824 (5)	0.3030 (7)	0.4874 (4)	0.0632 (13)	
H18	−0.2719	0.1908	0.5016	0.076*	
C19	−0.3424 (6)	0.4035 (10)	0.5456 (5)	0.0866 (19)	
H19	−0.3735	0.3600	0.5987	0.104*	
C20	−0.3552 (5)	0.5662 (10)	0.5243 (6)	0.088 (2)	
H20	−0.3955	0.6344	0.5636	0.106*	
C21	−0.3106 (6)	0.6341 (8)	0.4465 (6)	0.086 (2)	
H21	−0.3205	0.7468	0.4339	0.103*	
C22	−0.2511 (5)	0.5356 (6)	0.3871 (5)	0.0660 (14)	
H22	−0.2204	0.5803	0.3341	0.079*	
C23	0.2995 (4)	0.1765 (5)	0.5939 (3)	0.0380 (8)	
C24	0.3438 (5)	−0.0005 (5)	0.5889 (4)	0.0540 (12)	
H24A	0.3226	−0.0366	0.5203	0.065*	
H24B	0.2936	−0.0696	0.6254	0.065*	
N1	0.0045 (3)	0.2503 (4)	0.3896 (2)	0.0356 (7)	
N2	−0.1036 (5)	−0.0428 (5)	0.7741 (3)	0.0598 (11)	
O1	−0.1734 (3)	0.3120 (5)	0.2399 (2)	0.0654 (9)	
O2	−0.2013 (3)	0.0745 (4)	0.3449 (2)	0.0602 (9)	
O3	−0.1280 (5)	0.0831 (5)	0.7280 (4)	0.1015 (17)	
O4	−0.1194 (10)	−0.0537 (6)	0.8566 (4)	0.171 (4)	
O5	0.3599 (4)	0.2701 (4)	0.6549 (3)	0.0840 (13)	
S1	−0.16138 (10)	0.24019 (13)	0.33528 (8)	0.0465 (3)	
Br1	0.53169 (7)	−0.02967 (7)	0.64210 (6)	0.0930 (3)	

### Atomic displacement parameters (Å^2^)

**Table:**

	*U*^11^	*U*^22^	*U*^33^	*U*^12^	*U*^13^	*U*^23^
C1	0.041 (2)	0.0338 (19)	0.0347 (19)	0.0033 (16)	0.0131 (16)	0.0055 (16)
C2	0.068 (3)	0.046 (2)	0.041 (2)	0.009 (2)	0.012 (2)	0.0114 (19)
C3	0.078 (3)	0.035 (2)	0.063 (3)	0.005 (2)	0.035 (3)	0.015 (2)
C4	0.072 (3)	0.031 (2)	0.064 (3)	−0.0046 (19)	0.040 (3)	−0.0004 (19)
C5	0.048 (2)	0.039 (2)	0.050 (2)	0.0000 (17)	0.0229 (19)	−0.0033 (18)
C6	0.0388 (19)	0.0250 (18)	0.037 (2)	0.0017 (15)	0.0154 (16)	−0.0031 (16)
C7	0.0379 (19)	0.0241 (18)	0.0314 (18)	0.0009 (15)	0.0103 (15)	−0.0018 (14)
C8	0.0386 (19)	0.0281 (19)	0.0337 (18)	0.0002 (15)	0.0094 (15)	−0.0067 (15)
C9	0.038 (2)	0.028 (2)	0.039 (2)	−0.0018 (15)	0.0053 (16)	−0.0013 (15)
C10	0.0364 (19)	0.0233 (17)	0.043 (2)	0.0005 (15)	0.0108 (17)	−0.0048 (15)
C11	0.0287 (17)	0.033 (2)	0.038 (2)	−0.0034 (14)	0.0090 (16)	0.0015 (16)
C12	0.0358 (19)	0.033 (2)	0.044 (2)	0.0006 (16)	0.0136 (17)	−0.0053 (16)
C13	0.050 (3)	0.029 (2)	0.057 (3)	0.0020 (16)	0.017 (2)	−0.0053 (19)
C14	0.057 (3)	0.031 (2)	0.059 (3)	−0.0047 (18)	0.016 (2)	0.0098 (19)
C15	0.052 (2)	0.043 (2)	0.049 (2)	0.000 (2)	0.0239 (19)	0.008 (2)
C16	0.045 (2)	0.0307 (19)	0.044 (2)	0.0013 (17)	0.0143 (18)	−0.0052 (17)
C17	0.032 (2)	0.050 (3)	0.057 (3)	−0.0010 (17)	0.0001 (19)	−0.008 (2)
C18	0.052 (3)	0.069 (3)	0.069 (3)	0.001 (2)	0.015 (2)	−0.012 (3)
C19	0.059 (3)	0.102 (5)	0.105 (5)	−0.008 (3)	0.032 (3)	−0.036 (4)
C20	0.042 (3)	0.101 (6)	0.123 (6)	0.002 (3)	0.021 (3)	−0.049 (4)
C21	0.056 (3)	0.064 (4)	0.128 (6)	0.010 (3)	0.001 (4)	−0.033 (4)
C22	0.045 (3)	0.055 (3)	0.092 (4)	0.008 (2)	0.004 (3)	−0.009 (3)
C23	0.045 (2)	0.030 (2)	0.035 (2)	−0.0002 (17)	0.0018 (17)	−0.0032 (16)
C24	0.057 (3)	0.039 (2)	0.056 (3)	0.0065 (19)	−0.009 (2)	0.001 (2)
N1	0.0380 (16)	0.0322 (16)	0.0351 (16)	0.0039 (13)	0.0050 (13)	0.0071 (13)
N2	0.090 (3)	0.039 (2)	0.060 (3)	0.0112 (19)	0.038 (2)	−0.0005 (19)
O1	0.072 (2)	0.073 (2)	0.0417 (17)	0.0108 (18)	−0.0076 (15)	0.0061 (15)
O2	0.0532 (18)	0.0504 (19)	0.068 (2)	−0.0068 (14)	−0.0050 (16)	−0.0124 (15)
O3	0.160 (5)	0.047 (2)	0.127 (4)	0.025 (2)	0.097 (4)	0.008 (2)
O4	0.381 (12)	0.081 (3)	0.095 (4)	0.049 (5)	0.149 (6)	0.003 (3)
O5	0.090 (3)	0.051 (2)	0.084 (3)	0.0122 (18)	−0.037 (2)	−0.0277 (19)
S1	0.0421 (5)	0.0469 (6)	0.0439 (5)	0.0024 (5)	−0.0046 (4)	−0.0057 (5)
Br1	0.0538 (3)	0.0647 (3)	0.1580 (7)	0.0143 (3)	0.0183 (3)	−0.0032 (4)

### Geometric parameters (Å, º)

**Table:**

C1—C6	1.392 (5)	C14—H14	0.9300
C1—C2	1.403 (6)	C15—C16	1.374 (6)
C1—N1	1.412 (5)	C15—H15	0.9300
C2—C3	1.364 (7)	C16—N2	1.457 (5)
C2—H2	0.9300	C17—C18	1.379 (7)
C3—C4	1.368 (7)	C17—C22	1.387 (7)
C3—H3	0.9300	C17—S1	1.742 (4)
C4—C5	1.381 (6)	C18—C19	1.378 (8)
C4—H4	0.9300	C18—H18	0.9300
C5—C6	1.396 (6)	C19—C20	1.349 (11)
C5—H5	0.9300	C19—H19	0.9300
C6—C7	1.439 (5)	C20—C21	1.370 (10)
C7—C8	1.373 (5)	C20—H20	0.9300
C7—C23	1.475 (5)	C21—C22	1.378 (8)
C8—N1	1.406 (5)	C21—H21	0.9300
C8—C9	1.445 (5)	C22—H22	0.9300
C9—C10	1.332 (5)	C23—O5	1.197 (5)
C9—H9	0.9300	C23—C24	1.508 (5)
C10—C11	1.472 (5)	C24—Br1	1.907 (5)
C10—H10	0.9300	C24—H24A	0.9700
C11—C12	1.383 (5)	C24—H24B	0.9700
C11—C16	1.400 (5)	N1—S1	1.692 (3)
C12—C13	1.370 (5)	N2—O4	1.188 (6)
C12—H12	0.9300	N2—O3	1.198 (5)
C13—C14	1.380 (6)	O1—S1	1.420 (3)
C13—H13	0.9300	O2—S1	1.416 (3)
C14—C15	1.361 (6)		
			
C6—C1—C2	121.2 (4)	C16—C15—H15	120.1
C6—C1—N1	107.7 (3)	C15—C16—C11	122.3 (3)
C2—C1—N1	131.1 (4)	C15—C16—N2	116.7 (4)
C3—C2—C1	117.3 (4)	C11—C16—N2	121.1 (3)
C3—C2—H2	121.3	C18—C17—C22	120.4 (5)
C1—C2—H2	121.3	C18—C17—S1	119.9 (4)
C2—C3—C4	121.9 (4)	C22—C17—S1	119.6 (4)
C2—C3—H3	119.0	C19—C18—C17	120.3 (6)
C4—C3—H3	119.0	C19—C18—H18	119.9
C3—C4—C5	121.9 (4)	C17—C18—H18	119.9
C3—C4—H4	119.0	C20—C19—C18	118.8 (7)
C5—C4—H4	119.0	C20—C19—H19	120.6
C4—C5—C6	117.5 (4)	C18—C19—H19	120.6
C4—C5—H5	121.3	C19—C20—C21	122.0 (6)
C6—C5—H5	121.3	C19—C20—H20	119.0
C1—C6—C5	120.1 (4)	C21—C20—H20	119.0
C1—C6—C7	107.2 (3)	C20—C21—C22	120.0 (6)
C5—C6—C7	132.6 (4)	C20—C21—H21	120.0
C8—C7—C6	108.5 (3)	C22—C21—H21	120.0
C8—C7—C23	129.2 (3)	C21—C22—C17	118.4 (6)
C6—C7—C23	121.8 (3)	C21—C22—H22	120.8
C7—C8—N1	107.9 (3)	C17—C22—H22	120.8
C7—C8—C9	126.8 (3)	O5—C23—C7	120.4 (4)
N1—C8—C9	125.2 (3)	O5—C23—C24	121.4 (4)
C10—C9—C8	121.0 (3)	C7—C23—C24	117.9 (3)
C10—C9—H9	119.5	C23—C24—Br1	112.6 (3)
C8—C9—H9	119.5	C23—C24—H24A	109.1
C9—C10—C11	125.5 (3)	Br1—C24—H24A	109.1
C9—C10—H10	117.3	C23—C24—H24B	109.1
C11—C10—H10	117.3	Br1—C24—H24B	109.1
C12—C11—C16	116.1 (3)	H24A—C24—H24B	107.8
C12—C11—C10	119.8 (3)	C8—N1—C1	108.6 (3)
C16—C11—C10	124.0 (3)	C8—N1—S1	125.4 (2)
C13—C12—C11	121.9 (4)	C1—N1—S1	121.7 (2)
C13—C12—H12	119.0	O4—N2—O3	121.1 (4)
C11—C12—H12	119.0	O4—N2—C16	118.5 (4)
C12—C13—C14	120.3 (4)	O3—N2—C16	120.1 (4)
C12—C13—H13	119.8	O2—S1—O1	120.4 (2)
C14—C13—H13	119.8	O2—S1—N1	106.46 (17)
C15—C14—C13	119.6 (4)	O1—S1—N1	105.34 (19)
C15—C14—H14	120.2	O2—S1—C17	109.5 (2)
C13—C14—H14	120.2	O1—S1—C17	109.5 (2)
C14—C15—C16	119.8 (4)	N1—S1—C17	104.24 (17)
C14—C15—H15	120.1		
			
C6—C1—C2—C3	0.4 (6)	C17—C18—C19—C20	−0.8 (8)
N1—C1—C2—C3	−179.1 (4)	C18—C19—C20—C21	0.1 (9)
C1—C2—C3—C4	0.0 (7)	C19—C20—C21—C22	0.3 (9)
C2—C3—C4—C5	−0.7 (7)	C20—C21—C22—C17	0.0 (8)
C3—C4—C5—C6	1.0 (6)	C18—C17—C22—C21	−0.7 (7)
C2—C1—C6—C5	0.0 (6)	S1—C17—C22—C21	−179.3 (4)
N1—C1—C6—C5	179.6 (3)	C8—C7—C23—O5	−146.1 (4)
C2—C1—C6—C7	−178.3 (4)	C6—C7—C23—O5	42.6 (6)
N1—C1—C6—C7	1.3 (4)	C8—C7—C23—C24	39.2 (6)
C4—C5—C6—C1	−0.7 (5)	C6—C7—C23—C24	−132.1 (4)
C4—C5—C6—C7	177.1 (4)	O5—C23—C24—Br1	−25.8 (6)
C1—C6—C7—C8	−2.4 (4)	C7—C23—C24—Br1	148.9 (3)
C5—C6—C7—C8	179.6 (4)	C7—C8—N1—C1	−1.6 (4)
C1—C6—C7—C23	170.6 (3)	C9—C8—N1—C1	175.4 (3)
C5—C6—C7—C23	−7.4 (6)	C7—C8—N1—S1	−158.3 (3)
C6—C7—C8—N1	2.4 (4)	C9—C8—N1—S1	18.7 (5)
C23—C7—C8—N1	−169.8 (4)	C6—C1—N1—C8	0.2 (4)
C6—C7—C8—C9	−174.6 (3)	C2—C1—N1—C8	179.7 (4)
C23—C7—C8—C9	13.2 (6)	C6—C1—N1—S1	157.9 (2)
C7—C8—C9—C10	41.5 (6)	C2—C1—N1—S1	−22.6 (6)
N1—C8—C9—C10	−135.1 (4)	C15—C16—N2—O4	19.0 (8)
C8—C9—C10—C11	−172.1 (4)	C11—C16—N2—O4	−162.0 (7)
C9—C10—C11—C12	34.8 (6)	C15—C16—N2—O3	−154.8 (5)
C9—C10—C11—C16	−148.3 (4)	C11—C16—N2—O3	24.3 (7)
C16—C11—C12—C13	−0.1 (5)	C8—N1—S1—O2	−32.0 (3)
C10—C11—C12—C13	177.0 (4)	C1—N1—S1—O2	174.2 (3)
C11—C12—C13—C14	0.1 (6)	C8—N1—S1—O1	−160.9 (3)
C12—C13—C14—C15	0.3 (7)	C1—N1—S1—O1	45.2 (3)
C13—C14—C15—C16	−0.7 (7)	C8—N1—S1—C17	83.8 (3)
C14—C15—C16—C11	0.8 (6)	C1—N1—S1—C17	−70.1 (3)
C14—C15—C16—N2	179.8 (4)	C18—C17—S1—O2	22.6 (4)
C12—C11—C16—C15	−0.3 (6)	C22—C17—S1—O2	−158.8 (3)
C10—C11—C16—C15	−177.3 (4)	C18—C17—S1—O1	156.7 (4)
C12—C11—C16—N2	−179.4 (4)	C22—C17—S1—O1	−24.7 (4)
C10—C11—C16—N2	3.7 (6)	C18—C17—S1—N1	−91.0 (4)
C22—C17—C18—C19	1.1 (7)	C22—C17—S1—N1	87.6 (4)
S1—C17—C18—C19	179.6 (4)		

### Hydrogen-bond geometry (Å, º)

**Table:**

*D*—H···*A*	*D*—H	H···*A*	*D*···*A*	*D*—H···*A*
C2—H2···O5^i^	0.93	2.38	3.147 (5)	139
C2—H2···O1	0.93	2.38	2.957 (6)	120
C9—H9···O2	0.93	2.47	2.827 (5)	103
C10—H10···O3	0.93	2.37	2.730 (5)	103

Symmetry code: (i) *x*−1/2, −*y*+1, *z*−1/2.
